# Posterior Urethral Valves in a Healthy-Appearing Athletic Adult: A Case Report

**DOI:** 10.7759/cureus.66514

**Published:** 2024-08-09

**Authors:** Venus S Liu, Michael A Qureshi, Muhammad A Aziz

**Affiliations:** 1 Internal Medicine, Florida International University, Herbert Wertheim College of Medicine, Miami, USA

**Keywords:** chronic renal failure, congenital abnormalities, extremely delayed presentation, bilateral hydronephrosis, posterior urethral valve

## Abstract

Posterior urethral valves (PUV) are a congenital malformation of the male urethra where the posterior opening connecting to the bladder is covered by membranous folds. Most cases are diagnosed antenatally, with postnatal cases typically diagnosed and surgically repaired within the first years of life. Delayed presentation beyond infancy is rare, with presentation into adulthood being exceedingly rare, especially in the United States. We present a case of an 18-year-old healthy-appearing athletic male who presented with delayed presentation of PUV. This patient with no significant past medical history presented to the emergency room upon referral by his primary care physician, who denoted incidental findings of elevated blood pressure and acute renal failure at his annual physical examination. This led to further investigation, including a full renal blood workup, bilateral renal ultrasound, and voiding cystourethrogram, which revealed severe bilateral hydronephrosis, cortical thinning, and diverticula of the bladder, prompting a diagnosis of PUV. The patient underwent laser valve ablation surgery, although unfortunately, the surgery will prevent symptoms from progressing but is unlikely to reverse the current stage of chronic kidney damage. Even though delayed presentation of PUV is rare, it is important to recognize that patients may have a long history of renal complaints and may have normalized and internalized their symptoms. Physicians should take detailed and holistic medical histories and create a safe, non-judgmental environment to build rapport with young adult patients, ensuring early and effective medical intervention.

## Introduction

Posterior urethral valves (PUV) are a congenital malformation of the male urethra where the posterior opening connecting to the bladder is covered by membranous folds. The effects of urinary outflow obstruction on the bladder and kidneys in male neonates and infants will determine the prognosis for these patients; unrepaired obstruction can lead to chronic renal failure and even end-stage renal disease in childhood [1]. The reported incidence of PUV is estimated to be 1 in 8000 male live births. However, in the past 40 years, more advanced prenatal ultrasound screening, better medical support at birth and renal management, and early intervention have resulted in better outcomes [2]. Early intervention, such as catheter insertion and corrective surgery within the first years of life, can rapidly alleviate urethral obstruction and help normalize metabolic imbalances [3]. Therefore, delayed presentation beyond infancy is rare, and presentation in adulthood is exceedingly rare. Occasionally, some cases have been reported in adults without other medical comorbidities [4-6]. However, these cases are from Germany, Pakistan, and Brazil. Currently, in the United States, there is a lack of reports of late presentation of PUV in adults with no significant past medical history. Furthermore, these previous reports mainly focus on the possibility of late presentation of PUV and the importance of using voiding cystourethrography (VCUG) in adult male patients without further exploring the potential causes that lead to late presentation, such as psychosocial factors. Our case highlights a young, athletic, healthy-appearing patient's complete lack of awareness of the severity of his symptoms for years and the progression of renal failure without actively seeking medical treatments.

## Case presentation

An 18-year-old male with no significant past medical history presented to the emergency department (ED) on recommendation from his primary care provider due to elevated blood pressure of 155/100 mmHg during an annual wellness check and a subsequent abnormal creatinine level of 2.5 mg/dL (reference range 0.6-1.2 mg/dL). The patient was asymptomatic at the time; however, he has a long history of recurrent urinary symptoms dating back to childhood. The patient's symptoms initially began in the sixth grade, with episodes of dysuria and hematuria lasting for three days at a time, with one-to-three-month intervals free of symptoms. These symptoms were completely resolved in high school without intervention. However, by his senior year of high school, he had noted episodes of enuresis, increased urinary frequency, voiding 8-15 times, severe hesitancy, and straining to void. These symptoms had lasted for a year, but he did not seek medical care due to the on-and-off nature of the symptoms and the belief that they would resolve spontaneously, as they had in the past. Three weeks prior to the ED visit, he reported his first episode of dizziness and fatigue while playing basketball. He denied daytime urinary incontinence, constipation, or fecal incontinence. He had no known episodes of urinary tract infections and denied any history of sexual activity. He denied any family history of renal, bladder, other genitourinary, or psychiatric conditions.

**Figure 1:**
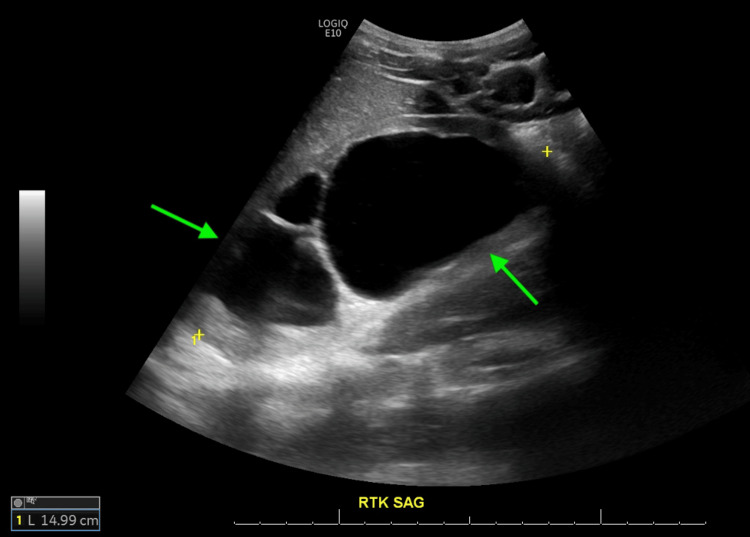
Left-sided renal ultrasound shows severe hydronephrosis with cortical thinning and the presence of echogenic material in renal pelvises, along with proximal ureters.

**Figure 2:**
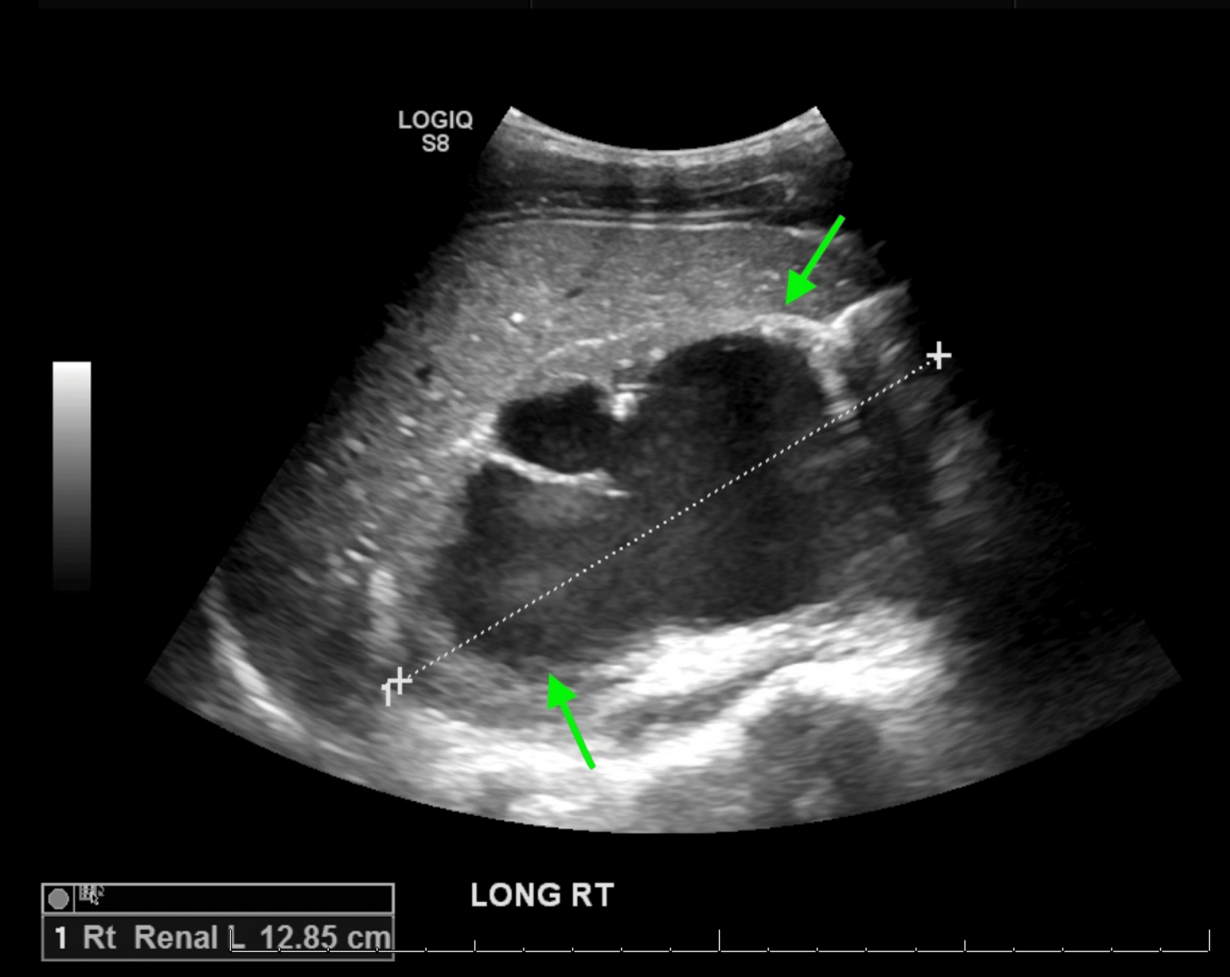
Right-sided renal ultrasound shows severe hydronephrosis similar to that on the left side.

On physical examination in the ED, the patient appeared to be a tall, athletic young adult in no acute distress. Abdominal examination revealed a soft, nondistended abdomen with normal bowel sounds and no organomegaly. The genitourinary exam was positive for pain with palpation over the suprapubic region without costovertebral angle tenderness. The neurologic examination noted no focal neurological deficits and normal sensory and motor function. The upper and lower extremities were non-edematous. ED laboratory results noted elevated parathyroid hormone of 92 pg/mL (reference range 10-60 pg/mL), cystatin-C of 1.79 mg/L (reference range 0.62-1.15 mg/L), potassium of 5.1 mmol/L (reference range 3.5-5 mmol/L), and creatinine of 2.6 mg/dL. The results of urinary analysis and sexually transmitted disease testing were negative. A Foley catheter was attempted four different times before final insertion in the ED, and it drained blood-tinged urine without frank blood or clots. Initial bilateral renal ultrasound noted severe bilateral hydronephrosis with cortical thinning and the presence of echogenic material in renal pelvises, along with proximal ureters and bladders, which may reflect infectious or proteinaceous debris (Figures 1, 2). The patient was admitted for further evaluation of acute renal failure.

During his hospitalization, an extensive workup was performed. Due to the patient's multiple episodes of enuresis, urinary frequency, and urinary hesitancy, the patient's symptoms were suggestive of overflow incontinence, possibly secondary to an undiagnosed congenital condition. Our team considered two likely etiologies, neurogenic versus structural blockage. To investigate possible structural etiologies, we conducted the voiding cystourethrogram (VCUG). To rule out possible spinal abnormalities, we performed a thorough neurological physical examination and thoracolumbar magnetic resonance imaging (MRI).

**Figure 3:**
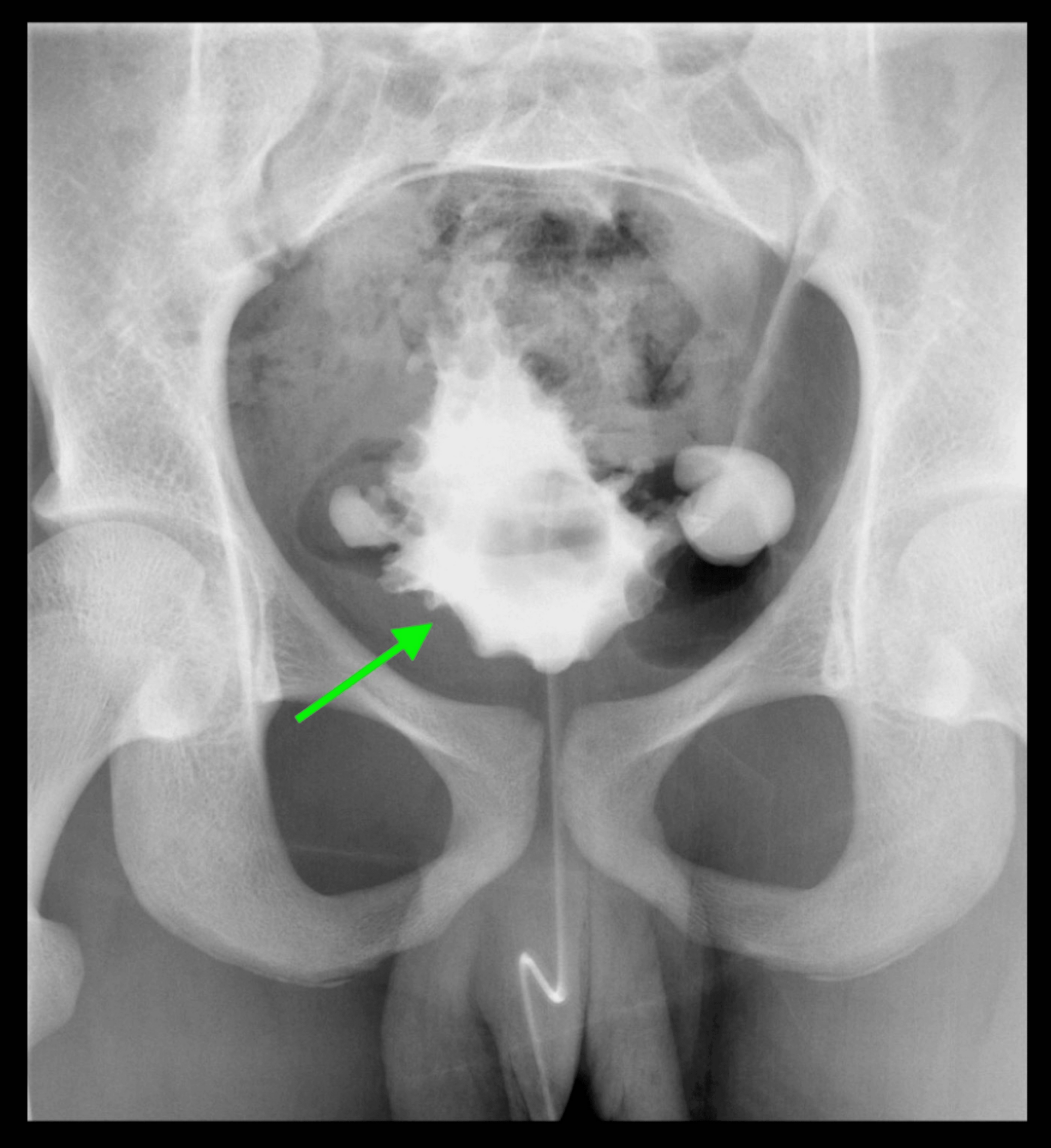
VCUG shows an abnormal bladder appearance; the bladder appears elongated with multiple trabeculations and diverticula. No obvious urethral stenosis, obstruction, or evidence of vesicoureteral reflux is noted. VCUG: voiding cystourethrography

**Figure 4:**
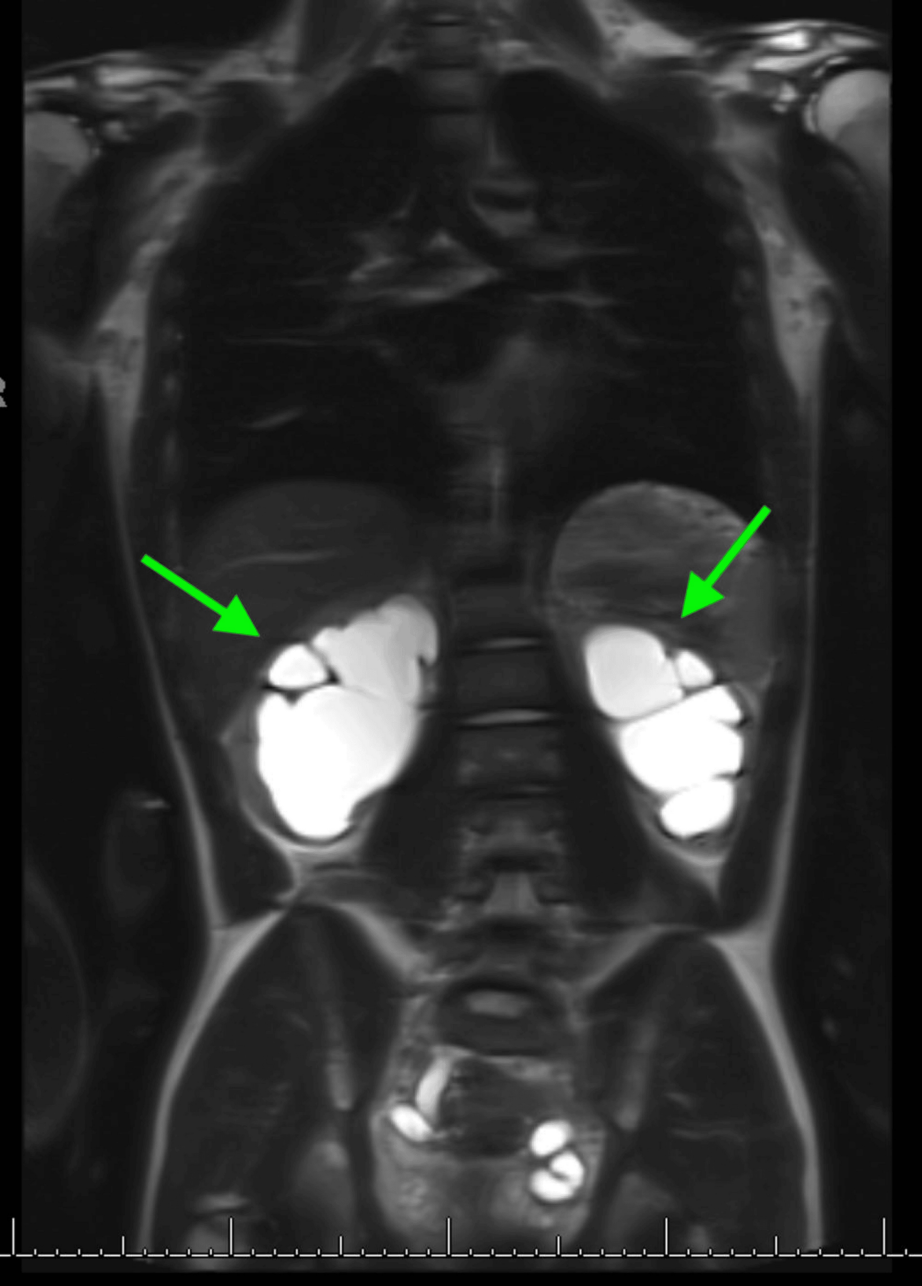
Normal MRI appearance of the thoracolumbar spine and no cord abnormality, tethering, or spinal dysraphism. It also shows a circumferentially thickened urinary bladder with massive hydroureteronephrosis. MRI: magnetic resonance imaging

Nephrology and pediatric urology were consulted. VCUG denoted an elongated bladder with multiple trabeculations and diverticula. There was no urethral stenosis or obstruction. The result provided evidence for bilateral hydronephrosis grade IV and chronic kidney damage. The characteristic "Christmas tree pattern" indicated evidence of PUV (Figure 3), although this is typically seen in younger males. After starting on 0.45% normal saline IV solution 1000 mL and amlodipine 5 mg one tab by mouth daily, blood pressure trended down, creatinine down to 2.3 mg/dL, and cystatin C down to 0.79 mg/L. A hemoglobin level of 11.2 g/dL (reference range 14-18 g/dL) and a hematocrit level of 34.1 L/L (reference range 41-50 L/L) revealed anemia likely secondary to long-standing kidney dysfunction. Renal duplex ultrasound demonstrated patent renal arteries with normal flow velocities and no evidence of stenosis or nonspecific diffuse bladder wall thickening. Thoracolumbar MRI noted a normal appearance of the thoracolumbar spine with no cord abnormalities, tethering, or spinal dysraphism; it also indirectly showed a circumferentially thickened urinary bladder with massive hydroureteronephrosis, consistent with PUV (Figure 4). The urology department planned and performed cystoscopy and laser valve ablation surgery within the same week.

Notably, near the end of his hospital stay, the patient became more communicative and expressed that he had only ever shared details of some of his symptoms with his mother and grandmother. He had never disclosed any of his urinary symptoms to medical providers previously due to fear of embarrassment, particularly feeling that his enuresis symptoms were especially shameful.

## Discussion

Before the widespread availability and use of prenatal ultrasound for the diagnosis of PUV in utero, late presentation of PUV was considered a favorable prognostic sign, correlating later presentation with lesser obstruction [7]. However, in modern times, such late-presenting cases are exceptionally rare due to the high prevalence of earlier treatment interventions. The most common historical signs that patients with late-presenting PUV initially describe include nocturnal enuresis, urinary frequency, and a history of urinary tract infections [8]. On physical examination, mild hypertension may be noted in less than 20% of cases. Common ultrasonographic findings include post-void residual bladder volume and hydronephrosis. VCUG remains the most definitive study for the diagnosis of PUV, showing characteristic tapering of the urethra. Other common VCUG findings include bladder trabeculation, vesicoureteral reflux, and diverticula [9]. The definitive treatment is surgical ablation of the valve. Most patients experience drastic improvements in symptoms following ablation, with improvements in voiding dysfunction [10]. However, not all patients have a complete resolution of symptoms after ablation. As many as 63% of patients may continue to experience some symptoms of voiding dysfunction following ablation [9]. As urologic management of PUV has evolved, many more patients are now surviving into adulthood. As detailed, these patients remain at increased risk of morbidity due to their condition and are therefore likely to benefit from long-term follow-up with adult providers [11].

Furthermore, past studies have shown that children commonly feel anxious and scared as they engage with medical professionals, and psychosocial distress may lead to a lack of cooperation and withdrawal regardless of whether they are involved in invasive or painful procedures or not [12]. Psychosocial barriers to care played a major role in our case's presentation and outcome. There is strong evidence showing that adolescents with urinary incontinence experience higher levels of psychosocial problems compared to those without urinary voiding dysfunction [13]. Specifically, adolescents who experience urinary incontinence often have poorer self-image, more negative perceptions of school, and more issues with peer relationships at school than those with normal urinary voiding control [13]. Our case highlights how psychosocial factors can impact medical outcomes. The patient's symptoms were personal, leading to a more severe and delayed presentation of the disease. This emphasizes the need for providers to build strong rapport with their patients, encouraging them to share personal symptoms that might otherwise be uncomfortable to discuss. By doing so, providers can reduce delays in care, prevent adverse outcomes, and offer better emotional support.

## Conclusions

Although multiple factors could have contributed to this patient's delayed presentation of PUV, early detection can lead to a better prognosis and less severe long-term renal failure. This case highlights the importance of rapport-building and detailed history-taking, even in healthy-looking young adults. Due to the rare nature of delayed PUV presentation, especially in the United States, it is crucial for medical providers to consider PUV as a potential diagnosis for overflow incontinence in young adults, not just in infants and toddlers. Additionally, it underscores the importance of recognizing psychological barriers and the potential chronic complications of this congenital condition into adulthood.
